# Interhemispheric Cerebral Blood Flow Balance during Recovery of Motor Hand Function after Ischemic Stroke—A Longitudinal MRI Study Using Arterial Spin Labeling Perfusion

**DOI:** 10.1371/journal.pone.0106327

**Published:** 2014-09-05

**Authors:** Roland Wiest, Eugenio Abela, John Missimer, Gerhard Schroth, Christian W. Hess, Matthias Sturzenegger, Danny J. J. Wang, Bruno Weder, Andrea Federspiel

**Affiliations:** 1 Support Center for Advanced Neuroimaging (SCAN), Institute for Diagnostic and Interventional Neuroradiology, University Hospital Inselspital and University of Bern, Bern, Switzerland; 2 Department of Neurology, Kantonsspital St. Gallen, St. Gallen, Switzerland; 3 Paul Scherrer Institute, Laboratory of Biomolecular Research, Villigen, Switzerland; 4 Department of Neurology, University Hospital Inselspital and University of Bern, Bern, Switzerland; 5 Department of Neurology, Ahmanson-Lovelace Brain Mapping Center, University of California Los Angeles, Los Angeles, California, United States of America; 6 Department of Psychiatric Neurophysiology, University Hospital of Psychiatry and University of Bern, Bern, Switzerland; University of Regensburg, Germany

## Abstract

**Background:**

Unilateral ischemic stroke disrupts the well balanced interactions within bilateral cortical networks. Restitution of interhemispheric balance is thought to contribute to post-stroke recovery. Longitudinal measurements of cerebral blood flow (CBF) changes might act as surrogate marker for this process.

**Objective:**

To quantify longitudinal CBF changes using arterial spin labeling MRI (ASL) and interhemispheric balance within the cortical sensorimotor network and to assess their relationship with motor hand function recovery.

**Methods:**

Longitudinal CBF data were acquired in 23 patients at 3 and 9 months after cortical sensorimotor stroke and in 20 healthy controls using pulsed ASL. Recovery of grip force and manual dexterity was assessed with tasks requiring power and precision grips. Voxel-based analysis was performed to identify areas of significant CBF change. Region-of-interest analyses were used to quantify the interhemispheric balance across nodes of the cortical sensorimotor network.

**Results:**

Dexterity was more affected, and recovered at a slower pace than grip force. In patients with successful recovery of dexterous hand function, CBF decreased over time in the contralesional supplementary motor area, paralimbic anterior cingulate cortex and superior precuneus, and interhemispheric balance returned to healthy control levels. In contrast, patients with poor recovery presented with sustained hypoperfusion in the sensorimotor cortices encompassing the ischemic tissue, and CBF remained lateralized to the contralesional hemisphere.

**Conclusions:**

Sustained perfusion imbalance within the cortical sensorimotor network, as measured with task-unrelated ASL, is associated with poor recovery of dexterous hand function after stroke. CBF at rest might be used to monitor recovery and gain prognostic information.

## Introduction

Widely distributed brain networks are involved in motor recovery after acute ischemic stroke. This has been evidenced by functional and effective connectivity analyses at rest and during tasks, the latter exemplified by changes in excitatory and inhibitory interactions between nodes of the somatomotor network [1]. Neuroimaging studies using blood-oxygen level dependent (BOLD) contrast have shown modulations of task-evoked activity in extended fronto-parietal and striato-cerebellar networks within the ipsi- and contralesional hemispheres during recovery of hand motor skills [2], [3], [4], [5], [6]. Such dynamics of BOLD contrast are the result of complex interactions within specific cortico-subcortical circuits and may be evoked also after a lesion of a remote subcortical node [7]. Inman et al. found reduced spontaneous resting-state connectivity in the ipsilesional hemisphere between the superior parietal cortex and both the primary sensorimotor (SM1) and supplementary motor area (SMA) during recovery in the subacute stage of stroke [8]. Two longitudinal studies disclosed significant spatial reorganizations of motor networks both in the ipsi- and contralesional hemisphere from early to late recovery phase [7], [9]. Implicated is the issue of interhemispheric balance which has been studied in the sensorimotor system of normal volunteers by Fox and Raichle [10]. They showed a bihemispheric coherence of somatomotor cortex, including medial motor areas, and secondary somatosensory association cortices in correlation maps relying on resting state BOLD. Instroke patients, disruption of functional connectivity in the somatomotor network correlated with motor impairment of upper extremity after stroke [11]. Finally, bihemispheric structural alterations were identified after stroke, reflected by increases in grey matter volume of the contralesional precuneus (PRE) and ipsilesional paralimbic anterior cingulate cortex (pACC). These structural patterns were positively correlated with recovery of motor function [12]. In sum, these studies suggest that functional and structural changes in both hemispheres are associated with motor recovery and, furthermore, the degree of interhemispheric balance may be of significance.

The most frequently employed imaging technique to map network activity is BOLD-fMRI [13]. Arterial spin labeling (ASL) offers advantages over BOLD-fMRI in applications where slowly varying changes in brain function are investigated [14]. The ASL signal is straightforward because it facilitates the direct quantification of cerebral blood flow (CBF). In comparison to the BOLD signal, it correlates well with the actual site of metabolism and neuronal involvement [15]. In cerebrovascular disease, ASL has been applied to investigate several issues such as perfusion changes, collateral flow, low-flow conditions and the effects of arterial stenosis [16], [17], [18], [19], [20].

Here, we examine the association between recovery of motor hand function relying on specific and standardized motor tasks and perfusion patterns, using serial ASL measurements in the early chronic phase after cortical ischemic stroke. We hypothesized that CBF patterns and their dynamics might allow drawing conclusions to precondition of recovery, implicated nodes of specific motor networks and interhemispheric CBF balance. We chose deliberately the time points for imaging at three and nine months in order to control for the duration of recovery processes. In addition, the three month examination should facilitate the comparison with studies dealing with CBF balance [21], [22], [23], [24].

## Materials and Methods

### Participants

We prospectively recruited patients at two comprehensive stroke centers (Departments of Neurology, University Hospital Bern and Kantonsspital St. Gallen, Switzerland) from January 01^th^, 2008 through July 31^th^, 2010. The study received ethical approval from both research centers (Ethikkommission des Kantons St. Gallen (EKSG), Kantonsspital St. Gallen, 9007 St. Gallen and Kantonale Ethikkommission Bern (KEK), 3010Bern, Switzerland) and all participants gave written informed consent before enrollment. Data from this cohort have been previously employed for a lesion analysis study [25]. Inclusion criteria were: (1) first-ever stroke, (2) clinically significant contralesional hand plegia or paresis as a main symptom, and (3) involvement of the pre-and/or postcentral gyri confirmed on diffusion-weighted (DWI) and fluid attenuated inversion recovery (FLAIR) scans. Exclusion criteria were: (1) aphasia or cognitive deficits severe enough to preclude understanding of study purposes, (2) prior cerebrovascular events, (3) significant stenosis (70–99% according to NASCET) or occlusion of the carotid and intracranial arteries on MR–angiography, (4) purely subcortical stroke, (5) other medical conditions interfering with task performance.

We studied 23 consecutive stroke patients (4 women, age range 41–78 y, mean age ± SD: 62.7±11.8 y). As a control group, we recruited 20 healthy participants from the local community (10 women, age range 55–75, mean age±SD: 63.6±6.5 y). Groups were matched for age (two-sample t-test: t (41)  = 2.4,p<.23) and (premorbid) handedness according to the Edinburgh Handedness Questionnaire (patients: median 82, range 65–100; controls: median 82, range 75–90, Mann-Whitney U-test U = 14.5, p<.35). For the patient group, behavioral data were recorded during the first week after stroke (baseline, days post-stroke, mean±SD: 5.6±3.6 d), at 3 months (93.2±8.3 d) and 9 months (277.3±13.2 d). All patient received neurorehabilitative treatment according to their clinical needs. In addition to the main measurement time-points, monthly control visits with assessment of motor hand function (see below) were performed to ensure appropriate intensity of neurorehabilitative treatment. Controls were tested at two visits separated by one month (29.5±1.3 d between examinations). ASL imaging was performed at 3 and 9 months post-stroke for patients, and repeated in 10 controls to assess the reliability of CBF measurements.

### Behavioral Data

#### Clinical and motor hand function assessment

Details on measurement procedures can be found in the supplementary materials. Stroke etiologies were classified according to the TOAST criteria [26]. Clinical stroke severity was assessed using the National Institutes of Health Stroke Scale (NIHSS) [27], [28]. Hand motor function was assessed with two outcome variables, grip force and dexterity. Grip force was measured by hand dynamometry (HD) with a Jamar Dynamometer [29]. Dexterous hand function was measured using the modified Jebsen-Taylor Test (JTT), a standardized quantitative assessment that consists of five timed subtests that simulate everyday activities [30], [31], [32]. For our current analysis, we relied on data from the JTT subtest "Picking Small Objects" (PSO), which consists of picking six common objects (2 paper clips, 2 bottle caps, 2 coins) and dropping them into an empty can as fast as possible. As previously shown by our group, PSO explains by far most of the longitudinal variance in JTT scores and allows accurate classification of patient subgroups [25]. The two motor tasks measure complementary aspects of hand motor function. Behaviorally, HD is performed with a simple power grip using the whole hand, whereas PSO necessitates successive precision grips (e.g. characterized by opposition of the thumb against one or two fingers) [33]. Neuroanatomically, each grip form is controlled by different cerebral networks: power grips are mainly controlled by the primary sensorimotor cortices, whereas precision grip control includes the premotor and posterior parietal cortices [34], [35].

### Imaging Data

Details on acquisition parameters and preprocessing algorithms for all image modalities can be found in supporting information S1. We summarize the main procedures below.

#### Acquisition

All images were acquired on a 3T Siemens Magnetom Trio (Erlangen, Germany) equipped with a 12-channel radiofrequency head coil. We measured CBF using the pulsed arterial spin labeling (PASL) technique [36], [37] and obtained high-resolutionT1-weighted MR images with a 3D Modified Driven Equilibrium Fourier Transform (MDEFT) sequence [38].

#### Preprocessing

High-resolution anatomical images were normalized to standard Montreal Neuroimaging Institute (MNI) space using an unified normalization-segmentation algorithm, resulting in normalized anatomical image as well as normalized grey matter (GM), white matter and cerebrospinal fluid tissue maps [39]. Binary lesion masks were used to exclude voxels within the lesion from the normalization process to avoid image distortions [40], [41]. Raw PASL images were first realigned to reduce movement artifacts. Quantified CBF flow time-series and average CBF maps for the entire acquisition were then calculated [42], [43]. Average CBF maps were coregistered to the anatomical image and normalized using the individual normalization parameters derived from the segmentation algorithm. Next, CBF maps were constrained to the GM by masking CBF images with normalized GM partitions that were binarized at a density threshold of 0.2. This empirical value assured that GM density values of at least 68% were included in the GM images. Finally, CBF images were smoothed with a 3D Gaussian kernel of 8×8×8 mm Full-Width at Half Maximum (FWHM) to reduce inter-individual anatomical differences. Before statistical analysis, all images were flipped such that the lesioned hemisphere was on the right. For quality control, we assessed the signal-to-noise ratio and test-retest reliability of CBF measurements for patients and controls before statistical analysis (see supporting information S1).

### Statistical analysis

#### Behavioral data

We used Fisher's exact test for count data. All behavioral variables were tested for normality using the Shapiro-Wilk test. Non-parametric tests were used where appropriate. Motor performance data were converted to z-scores using the mean and standard deviation of corresponding healthy control scores (HD: 36.0±12.0 kg, PSO: 5.9±1.2 s), such that lower z-scores represented lower motor performance. As in our previous work, patients that attained a PSO z-score of >-2.5 (p<.01, one-tailed) at Month 9 were empirically classified as successfully recovered (SR), all others as impaired recovered (IR) [25].

#### Imaging data

Lesion masks of patient subgroups were summed and binarized such that only voxels lesioned in ≥50% of the patients in each subgroup were retained. For voxel-wise analysis of CBF maps, we first performed a between-group comparison within the framework of the general linear model in SPM8 using a mixed-design analysis of variance (ANOVA) in order to assess the main effect of group (group factor with three levels: one for healthy controls, two for the repeated measurements of stroke patient subgroups at month 3 and month 9). Age and global mean CBF values were included as nuisance covariates. In order to account for stroke severity, all voxel-wise analyses were repeated after adjusting global mean CBF for total lesion volume using linear regression.

The resulting statistical parametric map was thresholded at p<.05, Family-Wise Error (FWE) corrected for multiple comparisons. Post-hoc unpaired t-tests were computed to compare cross-sectional between-group effects (patients at each exam against controls). Finally, paired t-test for longitudinal within-group effects, the main focus of this study, were computed (each patient subgroup separately). T-tests were calculated without equal variance assumptions. The resulting statistical parametric maps were explored using a threshold of p<.001(uncorrected), constrained to a cluster size of ≥ 50voxels. To assess the neuroanatomical distribution of lesions and significant CBF clusters, we used a cytoarchitectonic probabilistic atlas in MNI space (see supporting information S2).

To analyze changes of interhemispheric balance within the sensorimotor network in each subject, we extracted CBF values from functionally defined cortical regions of interest (ROI) as derived from our previous fMRI studies in healthy volunteers during tactile object manipulation [44], [45]. The rationale for doing so was that this task required fine-tuned sensory-guided finger movements, very similar to the PSO test used here to assess dexterous recovery and classify patient subgroups. These ROI included the dorsal premotor cortex (dPMC), supplementary motor area (SMA), paralimbic anterior cingulate cortex (pACC), primary motor cortex (M1), primary somatosensory cortex (S1), intraparietal sulcus (IPS), and superior precuneus (sPRE). Additionally, and ROI of the dorsolateral prefrontal cortex (dlPFC), which also participates in motor execution, was derived from an atlas of resting-state fMRI networks (http://findlab.stanford.edu/functional_ROIs.html). For details on ROI see table S1, supporting information S1. Laterality indices (LI) between hemispheres were calculated according to the standard formula [46]: 
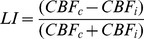
where *c* and *i* denote the contra- and ipsilesional hemisphere, respectively [36]. LI were calculated for each ROI individually, as well as across the whole network (by summing all individual ROI-LI), defined as “sensorimotor LI” throughout the paper.

## Results

### Behavioral data

According to our classification criterion, 17 patients were classified as SR and 6 as IR. Subgroup characteristics and motor examination results are summarized in Table 1. Proportions of male versus female patients (Fisher's exact test, p = .27, odds ratio [95%CI]: 3.5 [0.2–63.6]), and left versus right sided paresis (Fisher's exact test, p = .62, 0.3 [.01–4.7]) as well as mean age (Welch's t-test, t (41)  = 1.4, p = .36) were not statistically different between subgroups. The IR group showed a trend for higher NIHSS scores at Baseline (Wilcoxon rank sum test, W = 21.5, p = .07) and significantly higher scores at month 9 (W = 14, p<.02).

**Table 1:** Clinical characteristics of recovery subgroups.

	Successful Recovery(n = 17)			Impaired Recovery(n = 6)		
Gender(M/F)	15/2			4/2		
Affected Hand(L/R)	11/6			5/1		
Etiology (n)*	LA (5), CE (7), OC(1), UN(4)			LA (2), CE (3), OC (1)		
Age(y)	64.2±12.3(41–78)			59.0±10.3 (49–78)		
Lesion volume(cm^3^)	16.0±21.0 (5.3–23.5)			68.6±37.8 (35.8–121.2)		
	*Baseline*	*Month 3*	*Month 9*	*Baseline*	*Month 3*	*Month 9*
NIHSS	3.8±1.3	1.6±0.8	0.6±0.8	6.8±3.1	5.0±3.3	3.3±2.7
HD (kg)	21.2±14.1	36.5±11.9	41.9±10.5	2.0±12.5	23.0±17.0	33.2±17.2
HD (z-scores)	−1.2±1.2	0.0±1.0	0.5±0.9	−1.3±1.1	−1.1±1.4	−0.2±1.4
PSO (s)	10.2±9.2	7.4±3.5	6.5±2.0	27.4±14.6	17.1±13.5	10.9±3.3
PSO (z-scores)	−2.9±5.6	−1.2±2.9	−0.5±1.7	−18.3±11.7	−9.3±11.3	−4.1±2.8

Abbreviations: HD, hand dynamometry; NIHSS, National Institutes of Health Stroke scale; PSO, picking small objects. All values are mean ± SD (range), except for gender and affected hand (absolute numbers). *Etiology according to the TOAST criteria: LA, large artery arteriosclerosis; CE, cardioembolism; SO, small-vessel occlusion; OC, other determined cause; UN, undetermined cause.

**Figure 1:**
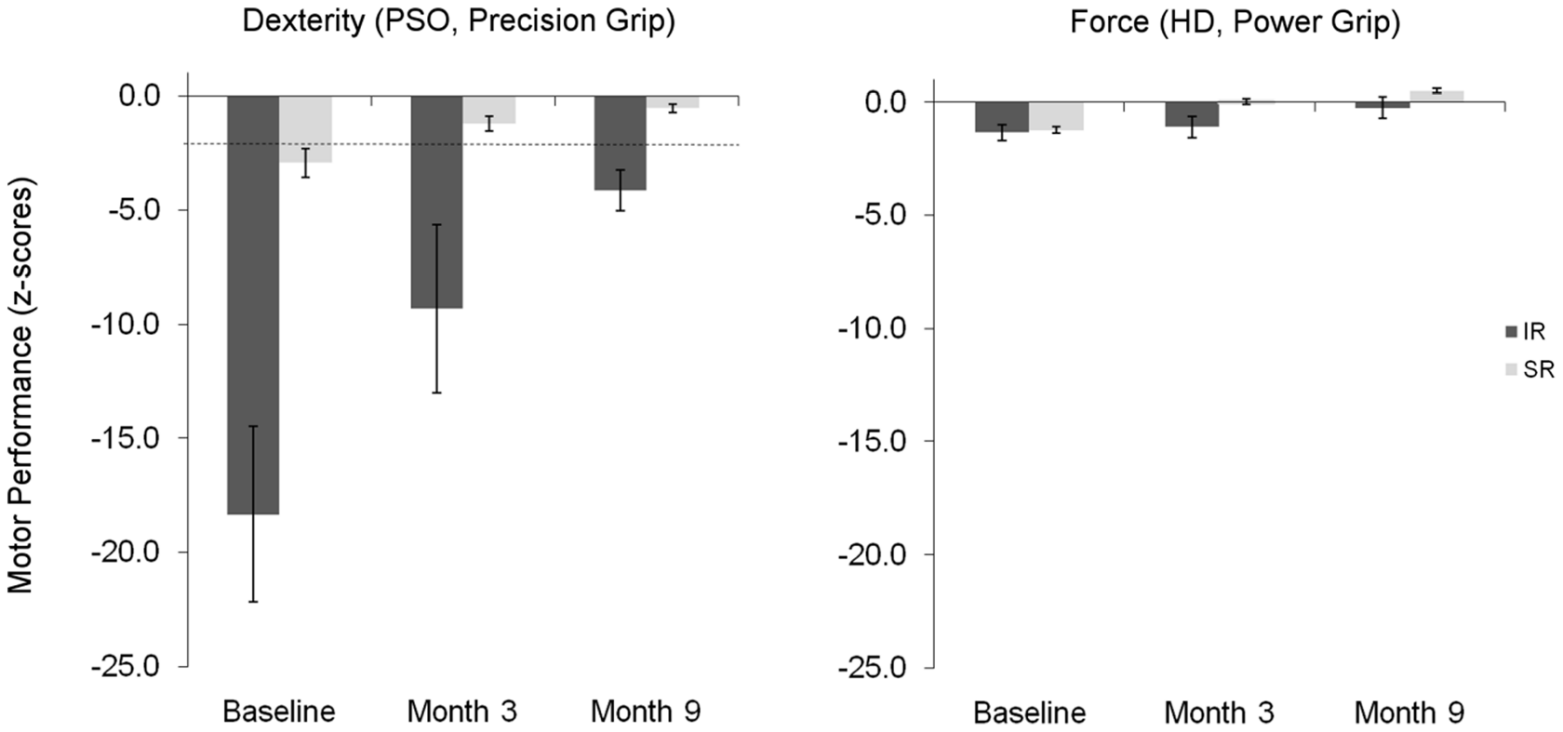
Time course of motor hand function in impaired (IR) and successful recovery (SR) subgroups. Left panel shows the recovery of dexterity, as measured with the picking small objects (PSO) task. The right panel shows the recovery of grip force, as measured with hand dynamometry (HD). Motor performance (y-axis) is given in z-scores task compared to healthy controls (lower scores indicate worse motor performance). Dashed line indicate thresholds for z = −2.5 (p<.01).

Concerning motor hand function, grip force (as measured by HD) and dexterity (as assessed with PSO) showed markedly different recovery time courses (Figure 1). Grip force between month 3 and month 9 was statistically different for both subgroups (Wilcoxon signed rank test for SR: W = 17, p < 0.01, for IR: W = 0, p<.05), but these changes occurred within the range of healthy control performance (Figure 1, right panel). Dexterity (PSO performance) showed no statistically significant change between examinations (SR: W = 42, p = .19, IR: W = 6, p = .43). SR patients performed at 3 months already within healthy control performance (p = .12), and IR patients remained impaired throughout examinations (both p<.001 versus healthy control performance) (Figure 1, left panel). At month 9 PSO and NIHSS had a significant negative correlation (Spearman's rank correlation, ρ = −0.45, p<0.05), in contrast to HD and NIHSS (ρ = −0.26, p = .22).

### Lesion data

Lesion distribution maps of both subgroups are shown in supporting information S2 and quantified using cytoarchitectonic maps in Table 2. Overall, patients in the IR group had a significantly higher lesion volume (Welch's t-test t = 4.1, p<0.02) than SR patients. Moreover, although in both groups the lesion core covered the primary sensorimotor cortices, the IR lesion map affected these areas more extensively, and included a large portion of the posterior parietal cortex, covering areas of IPS, supramarginal gyrus and parietal operculum. Higher lesion load in IR patients corresponds to our previous observations [25]. Additionally, we quantified lesion load on the cortico-spinal tract (CST) and superior longitudinal fascicle (SLF). Adjusting for total lesion volume, none of them showed a statistically significant difference across groups (both p>.1).

**Table 2:** Affected neuroanatomical areas in the lesion core of patient subgroups.

	Anatomical Region	Cytoarchitectonic Area	SR				IR			
			Vol%	x	y	z	Vol%	x	y	z
*Lesion core center of gravity*				33	−17	44		46	−16	30
*Affected areas*	Precentral gyrus	Area 4p	33.9	33	−31	45	80.6	37	−20	44
	Postcentral gyrus	Area 3a	19.9	30	−32	48	72.4	32	−30	40
		Area 3b	8.9	41	−21	48	7.0	42	−17	40
		Area 1	0.9	48	−21	55	72.4	50	−19	51
		Area 2	0.1	46	−24	49	56.4	45	−10	43
	Intraparietal sulcus	hIP2	0.0	-	-	-	73.9	46	−38	45
	Inferior parietal lobule	IPC (PF)	0.0	-	-	-	63.6	51	−17	28
		IPC (PFcm)	0.0	-	-	-	70.8	53	−23	35
		IPC (PFop)	0.0	-	-	-	100	56	−24	30
		IPC (PFt)	0.0	-	-	-	99.7	54	−30	44
	Parietal operculum	OP 1	0.0	-	-	-	100.0	53	−26	24
		OP 2	6.9	37	−22	28	100.0	38	−24	21
		OP 3	9.7	39	−19	27	100.0	44	−16	21
		OP 4	0.0	-	-	-	99.9	58	−13	19
	Cortico-spinal tract	CST	48.8	33	−16	43	56.5	45	−20	34
	Superior longitudinal fascicle	SLF	35.1	30	−21	28	86.2	31	−40	38

Abbreviations: hIP, human intraparietal sulcus; IPC (inferior parietal cortex), OP, operculum. Vol% indicates volume percent of each area covered by the lesion core map of each subgroup. Only areas damaged above 50% of their volume in any of the two groups are shown. Coordinates indicate the center of gravity of the lesion core in MNI space (x/y/z, in mm).

### Cross-sectional CBF differences between patients and healthy controls

In the ANOVA model, we identified overall between-group differences almost exclusively in the ipsilesional hemisphere, i.e. in SMA (Area 6), the lower M1 and S1 (Area 4p and 3b), the secondary somatosensory cortex (SII) and subregions of the inferior parietal cortex. ROI analyses on raw CBF values revealed that the overall differences corresponded to hypoperfusion relative to healthy controls. T-tests between patients and healthy controls at each time point revealed that most differences apparent at month 3 were reversible at month 9. As the results were expected with respect to the lesion topography, this analysis provided a quality control for the evaluation of the longitudinal analysis between IR and SR subgroups (see Fig. S1 and Table S2 in supporting information S2).

### Longitudinal CBF differences in patients with successful and impaired recovery

**Figure 2:**
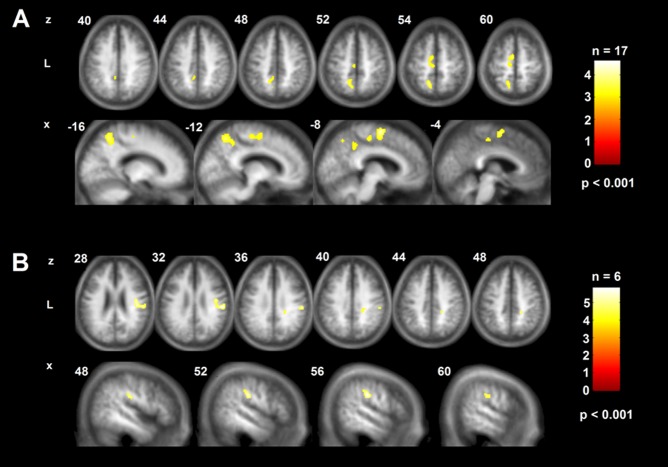
Longitudinal CBF differences between three and nine months for patients with successful and impaired recovery. A longitudinal decrease in supplementary motor area, paralimbic anterior cingulate cortex and superior precuneus is apparent in the SR group (panel A); whereas chronic sustained hypoperfusion in postcentral and supramarginal gyrus is found in the IR group (panel B). Maps are projected onto axial (z) and sagittal (x) slices of an average anatomical image of the complete patient cohort in neurological convention (L, left). The affected hemisphere is on the right side. Coordinates are given in MNI space (mm).

Voxel-wise comparisons revealed spatially distinct patterns of CBF change between exams and recovery subgroups (Figure 2). SR patients showed significant changes within two extended *contralesional* clusters involving midline motor nodes, i.e. the midline portion of the SMA, pACC and sPRE. Results were not qualitatively different when adjusting for total lesion volume (Fig. S2 in supporting information S3). In contrast, IR patients showed *ipsilesional* clusters of significant CBF change in the caudal portion of the postcentral gyrus, reaching into the inferior parietal lobule. In the SR group, CBF values in contralesional SMA/pACC and sPRE showed a reduction during recovery, whereas CBF values within the ipsilesional postcentral gyrus were unchanged. Conversely, CBF in the IR group remained extremely low in the ipsilesional postcentral gyrus and low in the contralesional midline motor nodes (Table 3). Again in contrast to the SR group, adjustment for total lesion volume lead to a disappearance of these effects, indicating a strong dependence of local CBF on stroke severity in IR patients (Fig. S2 in supporting information S3).

**Table 3:** Longitudinal changes of CBF in patient subgroups between 3 and 9 months.

Anatomical region	Cytoarchitectonic area (Vol%)*	x	y	z	Size	SR		IR		HC
						CBF Month 3	CBF Month 9	CBF Month 3	CBF Month 9	CBF
*Contralesional Clusters*										
Supplementary Motor Area Paralimbic Anterior Cingulate Cortex	Area6(87.1) n. a.	-9 -2	-12 0	61 46	589	61.5±12.1	48.7±11.2	37.7±13.2	36.4±8.7	64.6±22.1
Superior Precuneus	SPL7A(67.8)	-13	-57	54	237	47.6±12.5	34.8±17.8	36.81±11.1	39.91±10.6	50.1±18.6
*Ipsilesional Cluster*										
Inferior parietal lobule Postcentral gyrus	IPC(PFt)(21.4) Area3b(12.5)	54	-37	30	688	43.96±12.07	40.35±10.76	25.8±23.6	25.4±20.2	48.3±13.6

Abbreviations: CBF, cerebral blood flow (values are mean ± SD in ml/100 mg/min.); HC, healthy controls; n.a., not available. *Percent of cluster on each area.

### Interhemispheric CBF balance in the sensorimotor network

**Figure 3:**
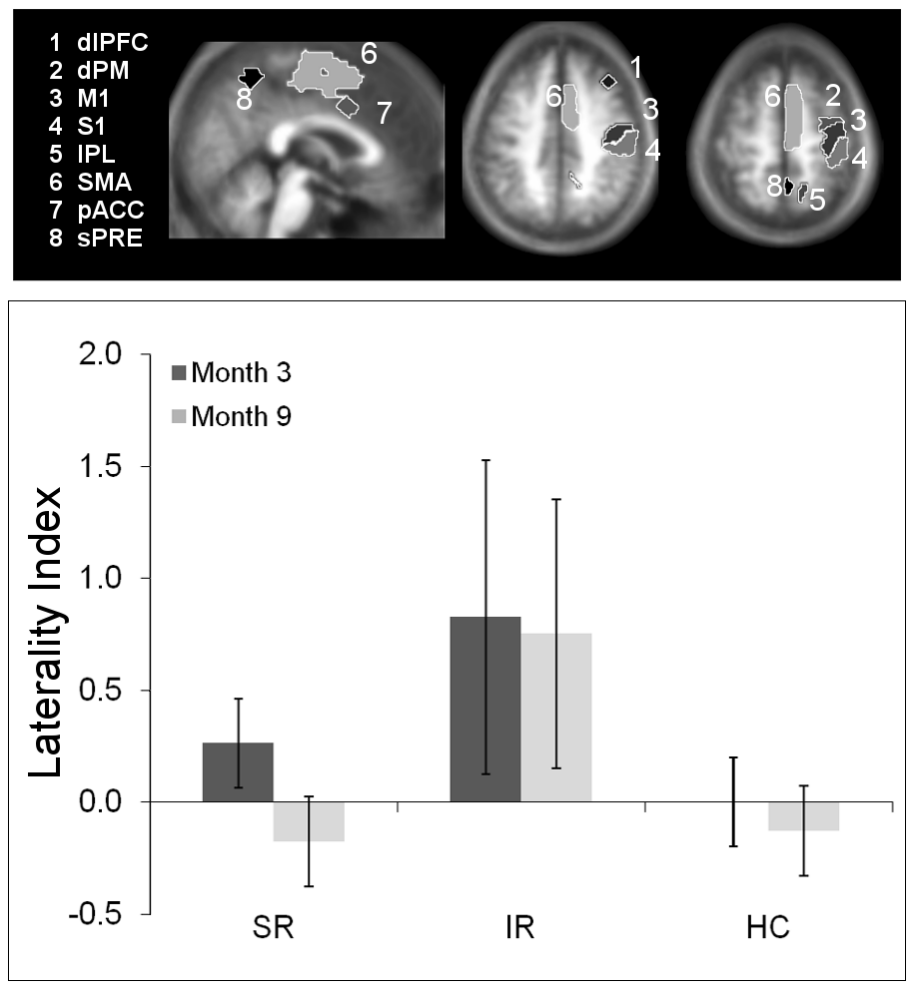
Longitudinal changes in sensorimotor laterality index in patient subgroups and healthy controls. Upper panel shows the set of regions-of-interest that defines the motor network. Only right hemispheric regions are shown for clarity. Lower panel shows sensorimotor laterality indices (LI) according to the defined network for patient subgroups and healthy controls at both examinations (for patients, Exam 1 denotes measurement at three months, Exam 2 nine months. For healthy controls, both examinations were one month apart). Positive values indicate contralesional lateralization. Note reversion of sensorimotor LI to lesioned hemisphere between examinations at 3 and 9 months for the successful recovered (SR) patients, whereas a persistent lateralization to the contralesional hemisphere is evident in impaired recovered (IR) patients. Healthy controls (HC) remain balanced. Bars indicate mean ±95% confidence intervals.

The time-course of the sensorimotor LI in the patient subgroups and its reproducibility in ten healthy volunteers with repeated ASL is shown in Figure 3. The detailed LIs according to regions and patient subgroups are specified in Fig. S3 in supporting information S3. The sensorimotor LI showed a trend level difference between all subgroups (both patient subgroups and healthy controls) at month 3 (Kruskal-Wallis test, K = 5.6, p = .06), but became significantly different at month 9 (K = 73, p<.05). Closer examination indicated that this occurred due to a significant reversal in laterality in the SR subgroup between time points (Wilcoxon rank sum test, W = 109.5, p<.05), whereas the IR group remained unchanged (W = 15.3, n.s.). The lower panel of Fig. 3 shows that balance returned to levels comparable to healthy controls in SR patients, whereas persisting imbalance is evident in the IR subgroup. Given the strong relationship between CBF and total lesion volume in this subgroup (see above), we further tested for a correlation between sensorimotor LI at 3 and 9 months and lesion volume. Rank correlation indeed revealed a significant dependence of the sensorimotor LI on stroke size in the IR subgroup (at 3 months: ρ = .78, p<.04, at 9 months: ρ = .81, p<.04), which was absent in SR patients (at 3 months: ρ = −.11, p<.45 at 9 months: ρ = −.29, p<.25). This indicates that above a given level of ischemic damage the capacity to re-balance interhemispheric CBF within the sensorimotor network was abolished in our cohort, impeding successful recovery.

## Discussion

This is a longitudinal study of a selected cohort of first-ever stroke patients with ischemic lesions involving the primary sensorimotor cortices, without connected upstream artery stenosis. All patients were examined at precisely defined time points post-stroke after 3 and 9 months. At this stage we observed a clinically dichotomized recovery, i.e.: i.) subjects who recovered mainly motor hand skill requiring precision grip in comparison with healthy volunteers (SR) and ii.) subjects retaining a significant chronic deficit in this regard (IR). Group differences were characterized exclusively by deficient precision grip, but not by power grip. Between groups, we verified simultaneously discordant courses of longitudinal CBF.

Apart from the primary sensorimotor cortex as a common denominator, the structural lesions in the IR-subgroup were more extended and involved the posterior parietal cortex, preferentially intraparietal sulcus, inferior parietal lobule and parietal operculum. Hypoperfused areas were located in this subgroup around the core lesion in the postcentral and supramarginal gyrus and persisted during the observation period. Similar reductions of perilesional CBF levels that are correlated with infarct size have already been reported in chronic stroke patients using ASL, indicating sustained hypoperfusion in the affected sensorimotor cortex [47]. In contrast, lesions in the SR subgroup were rather localized to the primary sensorimotor cortex and hypoperfusion had resolved in the stroke-affected hemisphere. However, significant reductions of CBF in the contralesional hemisphere along the evaluated time period could be identified within medially located nodes of the specific sensorimotor network: i.e. in the SMA, pACC and sPRE. Considering longitudinal CBF changes according to these subgroups, the preconditions of a double dissociation are fulfilled [51]: The IR-subgroup differed by an unchanged severe reduction of CBF between month 3 and 9 in the perilesional cortex, whereas SR-subgroup showed a significant reduction of CBF at month 9 confined to medial motor areas of the contralesional hemisphere. Overall, our data confirm the importance of restoring CBF in critical cortical network nodes for successful recovery of specific functions, as recently shown using ROI-analysis of MR-perfusion data in patients with cortical and subcortical stroke-associated aphasia [48], [49] and hemispatial neglect [50].

### Laterality indices within the sensorimotor network

There are few systematic studies on the subject of contrasting CBF-changes in both hemispheres after stroke, i.e. by the analysis of laterality indices, LIs [21], [22], [23], [24], [51]. Calautti et al. refined the computation of Cramer at al. by calculating a weighted LI of fMRI BOLD-response reflecting the changed bilateral distribution of activation fields evoked after motor stimulation of the recovered hand in the early chronic stage after a subcortical ischemic stroke. There were statistically significant correlations between maximum finger tapping and the LI for M1 and S1 after recovery. Marshall and coworkers computed LIs using fMRI and BOLD-response in subcortical ischemic stroke lesions during the immediate post-stroke and at early chronic stage, 3 to 6 months after stroke. They observed an evolution of activation in the sensorimotor cortex from early contralesional activity to late ipsilesional activity during movement of the paretic hand following regained function. Both studies admit only indirect inferences for CBF analysis, since they were possibly confounded by the influence of complex interhemispheric neuronal interactions of mutual interhemispheric inhibition, by disinhibition and the relativity of the BOLD signal [1]. BOLD responses are dependent on the severity of motor impairment as well as on CBF decreases at rest. Thus, task-related increases of the BOLD signal fMRI studies during recovery, as well as their reductions to physiological levels after 6–12 months may be partially addressed to CBF changes. Overall, longitudinal normalization of the BOLD signal is associated with good outcome [4], [52], [53], [54].

We complemented these studies in several regards: i.) evaluation in cortical strokes with a common, overlapping lesion site in the primary motor and sensory cortices, ii.) examination at rest, iii.) unbiased quantification of CBF by ASL instead of relative BOLD signal changes and iv.) analysis in cortical nodes of specific sensorimotor networks as previously identified by a sensorimotor fMRI activation study [44]. In healthy controls there was reproducible, stable balance within narrow margins over time within the nodes of sensorimotor circuits. Patients revealed a considerable variance of laterality indices across the time span. In the IR-subgroup the sensorimotor LI remained unchanged and shifted to the contralesional hemisphere. In the SR-subgroup the sensorimotor LI indicated a significant reversion to the lesioned hemisphere while the mean CBF decreased in the contralesional medial motor nodes. The crucial observation of an augmentative CBF dynamic is thus recognizable only in the SR-group: Changes of CBF and associated LIs may reflect ongoing neuronal reorganization associated with recovery. These findings support the significance of the image analysis as the areas of decreased CBF in both subgroups have been shown to be part of the constituents of sensorimotor networks distributed over both hemispheres. In contrast, the more extensive lesions with sustained poor perfusion, as exemplified by the IR-subgroup, seem to hinder the restitution of favorable interhemispheric balance. Indeed, the strong positive correlations between contralesional CBF shift (positive LI) and lesion volume in the IR subgroup indicate that above a certain threshold of ischemic damage longitudinal CBF adjustments cannot occur any longer. Interestingly, loss of interhemispheric CBF dynamics within the sensorimotor network does not preclude substantial gains in motor performance during recovery in the IR group (see Figure 1), but rather seems to provide an upper bound to motor performance. One explanation for this apparent paradox might be the recruitment of additional neural circuits that might provide compensatory, but coarse and ineffective behavioral output. Indeed, we have recently shown that extensive grey matter remodeling occurs in subcortical and cortical nodes of a basal ganglia-dorsolateral prefrontal network in IR-type patients, i. e. outside the "canonical" cortical motor loop, and the relationship between this phenomenon and CBF lateralization remains to be explored [55].

### Functional implications

Dynamic changes of CBF in the contralesional medial motor nodes of SR patients might represent a set point adjustment to reinstate interhemispheric balance with homologous motor nodes of the primarily lesioned hemisphere. This might be paralleled by diminished recruitment of these structures in the contralesional hemisphere, which in part subserve focused attention and motor execution [2], [11]. In this respect the pACC, a structure connected also to the dorsolateral prefrontal cortex, plays an important role in monitoring motor control, sensory perception, cognitive function and attention [56], [57]. The superior precuneus cluster identified in our study lies ventral to the superior parietal sulcus just above the subparietal sulcus. It has bilateral and reciprocal cortico-cortical connections with the adjacent posteromedial cortex, providing an anatomical basis for their functional coupling [58]. Beyond the parietal lobe, extensive connections exist between the superior precuneus and the dorsal premotor area, the supplementary motor area (SMA) and the anterior cingulate cortex [59], [60]. The posteromedial parietal cortex acts with the lateral parietal areas in elaborating information about egocentric spatial relations for body movement control (motor imagery), voluntary attention shift and mental imagery tasks. The lateral aspect of the posterior parietal cortex, the posterior parietal lobe and inferior posterior lobule are involved in controlling spatial aspects of motor behavior, e.g. during sensory guided finger movements [61]. By virtue of its connectivity, the superior precuneus is involved into the execution or preparation of spatially guided motor behavior, such as pointing and reaching, and in particular in coordination of both upper limbs during task execution [62]. Interestingly, a study by Marshall et al. has recently shown that early BOLD activity in precuneus and posterior parietal cortex during hand movements might be predictive for later recovery [46].

### Limitations

A few limitations must be kept in mind when interpreting our results. Sample sizes are small, especially for the IR subgroup, and thus generalization is difficult based on our results. However, this subgroup represents in our view an interesting and generally underrepresented subset in CBF studies, exhibiting hypoperfusion in the absence of (significant) arterial stenosis. The clarification of its underlying pathophysiological mechanisms remains a challenge for future studies of this peculiar association [47]. One methodical limitation to consider is that CBF quantification was done under the assumption that T1 relaxation of blood be constant over the whole brain. This is clearly an oversimplification which might cause biased CBF estimates in lesioned brain areas. We therefore included explicit lesion masks during CBF reconstruction to mitigate this bias. Also, the ASL sequence we used does not cover the cerebellum, and thus, we could not evaluate the well-known diaschisis effects and its role for recovery [63]. Furthermore, conclusions from ASL studies allow no direct comparison with activation studies performed with BOLD fMRI [47].

## Conclusions

ASL measurements during rest are capable to delineate subtle CBF-changes within nodes of specific large scale networks during the early chronic phase after a sensorimotor stroke. Such changes related to hand motor skill as shown by voxel-based analysis may exhibit an asymmetric pattern due to interhemispheric imbalance. Reversion of the laterality indices underlying this pattern in favor of the lesioned hemisphere goes along with recovery. Of note, the time course of recovery processes, clearly outlasts the first three months post stroke, indicating that not only acute [64], [65], but also early and late chronic measurements of cerebral hemodynamics are important to understand stroke recovery. The data provide us with a method to monitor recovery of specific motor hand functions and to evaluate the interrelation between chronic sustained hypoperfusion, infarct size and BOLD response.

## Supporting Information

Supporting Information S1 **Contains supplementary methods.** These include a description of the behavioral testing procedures, the MR data acquisition and processing and magnetic resonance image acquisition parameters.
(DOCX)

Supporting Information S2 **Describes the main findings of cross-sectional comparisons of CBF effects in patients versus healthy controls.**
(DOCX)

Supporting Information S3 **Describes the main findings in interhemispheric balance changes and the detailed LIs according to regions and patient subgroups.**
(DOCX)
