# Supplementary material for: Interhemispheric Cerebral Blood Flow Balance during Recovery of Motor Hand Function after Ischemic Stroke—A Longitudinal MRI Study Using Arterial Spin Labeling Perfusion
Source: PLoS One. 2014 Sep 5;9(9):e106327. doi: 10.1371/journal.pone.0106327 (PMC4156327; doi:10.1371/journal.pone.0106327)
Supplement: Supporting Information S1 — Contains supplementary methods. These include a description of the behavioral testing procedures, the MR data acquisition and processing and magnetic resonance image acquisition parameters. (DOCX) [file pone.0106327.s001.docx]

# Supporting information S1

**Behavioral Assessment**

All behavioral measurements were made by the same author (EA).

*Hand dynamometry*

A Jamar dynamometer was used to measure maximal grip force (i.e. power grip) over three trials, alternating between hands [1]. The dynamometer was held in front of the subject with the elbow flexed at 90° and slightly abducted to avoid contact with the trunk. Subjects were instructed to squeeze the lever of the apparatus as strongly as possible without extending the arm. An investigator supported the dynamometer such that the participant could comfortably exert a power grip without the need to additionally stabilize the dynamometer. Values were recorded in kg.

*Dexterous hand function*

Dexterity was measured for both hands using the modified Jebsen-Taylor Test (mJTT), a standardized quantitative assessment of hand function that consists of five timed subtests intended to simulate everyday activities, e.g. turning pages, eating using a spoon [2]. The test is scored by adding the seconds needed to complete each subtest (higher values indicate worse performance); standard performance norms are available from the original publication for both sexes and for different age groups [2]. Several studies have used this test to assess hand function in stroke [3-6]. We applied the mJTT with custom made materials according to the original instructions. Specifically, for the Picking Small Objects task, two paper clips, two bottle caps and two coins were positioned on the same side as the tested hand, touching a wooden board. The subject had to pick each object, starting with the one farthest away and put them into a can positioned at the body midline. The task was performed in mirror-symmetric fashion for both the contra- and ipsilesional hand.

**Imaging Data Acquisition**

*Anatomy: T1 Parameters*

The optimized acquisition parameters included: repetition time TR = 7.92 ms, echo time TE = 2.48 ms, flip angle = 16°, inversion with symmetric timing (inversion time 910 ms), 256 × 224 x 176 matrix points with a non-cubic field of view (FOV) of 256 mm × 224 mm x 176 mm, yielding a nominal isotropic resolution of 1 mm^3^ (i.e. 1mm × 1mm × 1mm), fat saturation, 12 min. total acquisition time. Identical prescription of MR images was achieved by use of the Siemens auto-align sequence that automatically sets up consistent slice orientation based on a standard MRI atlas.

*CBF: Pulsed ASL Parameters*

TR/TE = 4000/11ms , FOV = 230mm^2^, matrix size = 64x64, (the isocenter of the readout slice was 90mm above labeling plane), Bandwidth 3005 Hz/Pixel and flip angle (FA = 90°), time constants TI1 = 700 ms; TI2=1400 ms Fourteen axial slices (EPI readout, 3.4mm in-plane resolution, 6mm slice thickness and 3.0mm gap) were positioned parallel to the bi-commissural axis, and were shifted in z-direction to cover the primary motor area of each subject. A total of 80 images, i.e. 40 pairs of label and control images were recorded in less than 6 min. The sequence was recorded with 3D PACE (Siemens Erlangen, Germany) to enable prospective motion correction.

**Imaging Data Preprocessing**

*Anatomy: Normalization and Cost-Function Masking*

Anatomical data were normalized using SPM8 (<http://www.fil.ion.ucl.ac.uk/spm/software/>) for MATLAB. The SPM8 framework uses a set of affine (linear) and non-linear transforms to match an individual image (e.g. T1 scan) to a stereotactic template. Mismatch of images is quantified as the sum of the squared differences between the image voxel intensities, i.e. a "cost function", that is reduced during image registration [7]. While the affine part is relative insensitive to the presence of focal lesions, the non-linear estimation of the algorithm can lead to distortion of the damaged area and the surrounding tissue, usually resulting in reduced lesion volumes [7, 8]. This remains true for most real-world scenarios, even if newer normalization and registration algorithms perform well with simulated lesions [9]. It is therefore recommended to exclude the damaged voxels during the normalization process, in order to reduce their impact on cost function minimization [7]. To accomplish this, we used a "cost function masking" procedure with explicit binary lesion masks, as previously described [10]. First, all lesions were manually segmented by one author (EA) in native space onto diffusion weighted images (DWI) acquired at the acute phase (baseline) using MRIcron (<http://www.cabiatl.com/mricro/mricron/index.html>), yielding binary lesion images. The advantage of using baseline DWI scans is twofold: they provide superior contrast for the identification of the acute ischemic lesion (compared to T1 or T2-weigthed images), and acute DWI lesion volumes have an excellent correlation with chronic stroke lesion volumes [11]. To avoid bias, lesion segmentation was performed without reference to the results of the behavioral or CBF data analysis. Lesion images were smoothed with a 3D Gaussian kernel with 8.0 mm full-width at half maximum, inverted and binarized at a threshold of 0.1 to yield the final lesion masks [7]. DWI scans and lesion masks were then co-registered to the T1-images from Month 3. Finally, co-registered lesion masks and T1-images were simultaneously spatially normalized to Montreal Neurological Institute (MNI) stereotaxic space using the unified segmentation algorithm in SPM8 and resampled to 2.0 mm^3^ isotropic resolution.

*CBF: quantification*

Quantification of CBF flow time series was achieved using in-house scripts for MATLAB and was based on the equation:

$$CBF=\left(\frac{\lambda\cdot\Delta M}{2\cdot\alpha\cdot M_{0}\cdot Tl_{1}}\right)\cdot e^{Tl_{2}/Tl_{b}}$$

The timing constants Tl1 and Tl2 were set to 700ms and 1400 ms tagging duration = 700 ms, blood/tissue water partition coefficient λ = 0.9 [g/ml] and tagging efficiency assumed to be α = 0.95. For 3.0T the decay time for labeled blood T1b = 1650 ms, M0 are the equilibrium brain tissue magnetization images [12, 13]. ∆M was calculated by subtraction of all realigned label and control images [14].

*ASL Data: Estimation of Signal-to-Noise Ratio (SNR) and Test-Retest Reliability*

For each subject the global CBF signal averaged across all GM voxels was extracted. The individual SNR was calculated as the ratio of the temporal mean of the GM and its standard deviation. The mean SNR and its standard deviation were calculated across all subjects. To test the reliability of the repeated ASL measurements, correlations between global and regional CBF values were calculated for the data of 10 control subjects with two measurements. Specifically, we calculated the correlations of global mean CBF and GM-specific CBF between time-points (see Supplemental Results below).

**Neuroanatomical Localization and Functional Regions of Interest**

To provide a precise assignment of CBF and lesion effects to underlying structural neuroanatomy we used the Jülich cytoarchitectonic atlas, incorporated in the SPM Anatomy Toolbox (freely available at <http://www.fil.ion.ucl.ac.uk/spm/ext/#Anatomy>). The atlas consists of cytoarchitectonic probability maps of cortical and subcortical areas in MNI space, generated with an observer-independent procedure from 10 post-mortem brains of neurologically healthy patients. Details on atlas generation and MATLAB implementation can be found in [15, 16]. The toolbox allows the quantification of overlap between e.g. a significant CBF cluster and the maximum probability map of a given cytoarchitectonic area (e.g. the primary motor cortex, Area 4a/4p), thus providing quantitative neuroanatomical information rather than (subjective) assignment based on macroscopic landmarks [17].

For region of interest (ROI) analysis, we used clusters derived from fMRI studies of tactile object manipulation, as described in the main text. ROI size and centers of gravity are given in Table S1 below.

**Table S1. ROI Details**

| **Name** | **Abbreviation** | **Number of voxels** | **Center of Gravity (MNI Coordinates)*** | | |
| --- | --- | --- | --- | --- | --- |
|  |  |  | *x* | *y* | *z* |
| Dorsolateral prefrontal cortex | dlPFC | 266 | 38 | 26 | 44 |
| Dorsal premotor cortex | dPMC | 271 | 32 | -6 | 66 |
| Supplementary motor area | SMA | 92 | 2 | -6 | 70 |
| Paralimbic anterior cingulate cortex | pACC | 53 | 2 | 0 | 48 |
| Primary motor cortex | M1 | 1323 | 40 | -16 | 54 |
| Primary somatosensory cortex | S1 | 947 | 48 | -26 | 50 |
| Intraparietal sulcus | IPS | 25 | 26 | -60 | 56 |
| Superior precuneus | sPRE | 76 | 2 | -56 | 60 |

* For right hemisphere ROI. Left hemisphere ROIs have mirror-symmetric coordinates (negative x-coordinates).

# Supplemental References

[1] Mathiowetz V, Weber K, Volland G, Kashman N. Reliability and validity of grip and pinch strength evaluations. J Hand Surg Am. 1984 Mar;9(2):222-6.

[2] Jebsen RH, Taylor N, Trieschmann RB, Trotter MJ, Howard LA. An objective and standardized test of hand function. Archives of physical medicine and rehabilitation. 1969 Jun;50(6):311-9.

[3] Sunderland A, Bowers MP, Sluman SM, Wilcock DJ, Ardron ME. Impaired dexterity of the ipsilateral hand after stroke and the relationship to cognitive deficit. Stroke; a journal of cerebral circulation. 1999 May;30(5):949-55.

[4] Sunderland A. Recovery of ipsilateral dexterity after stroke. Stroke; a journal of cerebral circulation. 2000 Feb;31(2):430-3.

[5] Wetter S, Poole JL, Haaland KY. Functional implications of ipsilesional motor deficits after unilateral stroke. Archives of physical medicine and rehabilitation. 2005 Apr;86(4):776-81.

[6] Chestnut C, Haaland KY. Functional significance of ipsilesional motor deficits after unilateral stroke. Archives of physical medicine and rehabilitation. 2008 Jan;89(1):62-8.

[7] Brett M, Leff AP, Rorden C, Ashburner J. Spatial normalization of brain images with focal lesions using cost function masking. NeuroImage. 2001 Aug;14(2):486-500.

[8] Andersen SM, Rapcsak SZ, Beeson PM. Cost function masking during normalization of brains with focal lesions: still a necessity? NeuroImage. 2010 Oct 15;53(1):78-84.

[9] Ripolles P, Marco-Pallares J, de Diego-Balaguer R, Miro J, Falip M, Juncadella M, et al. Analysis of automated methods for spatial normalization of lesioned brains. NeuroImage. 2012 Apr 2;60(2):1296-306.

[10] Abela E, Missimer J, Wiest R, Federspiel A, Hess C, Sturzenegger M, et al. Lesions to primary sensory and posterior parietal cortices impair recovery from hand paresis after stroke. PLoS One. 2012;7(2):e31275.

[11] Lovblad KO, Baird AE, Schlaug G, Benfield A, Siewert B, Voetsch B, et al. Ischemic lesion volumes in acute stroke by diffusion-weighted magnetic resonance imaging correlate with clinical outcome. Annals of neurology. 1997 Aug;42(2):164-70.

[12] Wang J, Aguirre GK, Kimberg DY, Roc AC, Li L, Detre JA. Arterial spin labeling perfusion fMRI with very low task frequency. Magn Reson Med. 2003 May;49(5):796-802.

[13] Federspiel A, Muller TJ, Horn H, Kiefer C, Strik WK. Comparison of spatial and temporal pattern for fMRI obtained with BOLD and arterial spin labeling. J Neural Transm. 2006 Oct;113(10):1403-15.

[14] Wang J, Alsop DC, Song HK, Maldjian JA, Tang K, Salvucci AE, et al. Arterial transit time imaging with flow encoding arterial spin tagging (FEAST). Magn Reson Med. 2003 Sep;50(3):599-607.

[15] Eickhoff SB, Heim S, Zilles K, Amunts K. Testing anatomically specified hypotheses in functional imaging using cytoarchitectonic maps. NeuroImage. 2006 Aug 15;32(2):570-82.

[16] Eickhoff SB, Stephan KE, Mohlberg H, Grefkes C, Fink GR, Amunts K, et al. A new SPM toolbox for combining probabilistic cytoarchitectonic maps and functional imaging data. NeuroImage. 2005 May 1;25(4):1325-35.

[17] Amunts K, Zilles K. Advances in cytoarchitectonic mapping of the human cerebral cortex. Neuroimaging Clin N Am. 2001 May;11(2):151-69, vii.
