# Supplementary material for: Interhemispheric Cerebral Blood Flow Balance during Recovery of Motor Hand Function after Ischemic Stroke—A Longitudinal MRI Study Using Arterial Spin Labeling Perfusion
Source: PLoS One. 2014 Sep 5;9(9):e106327. doi: 10.1371/journal.pone.0106327 (PMC4156327; doi:10.1371/journal.pone.0106327)
Supplement: Supporting Information S2 — Describes the main findings of cross-sectional comparisons of CBF effects in patients versus healthy controls. (DOCX) [file pone.0106327.s002.docx]

# Supporting information S2

**Cross-sectional Findings**

Figure S1 and Table S2 summarize the main findings of cross-sectional comparisons of rCBF effects in patients versus healthy controls. See main manuscript for details.

**Figure S1. Cross-sectional CBF differences between healthy controls and patients.**

**
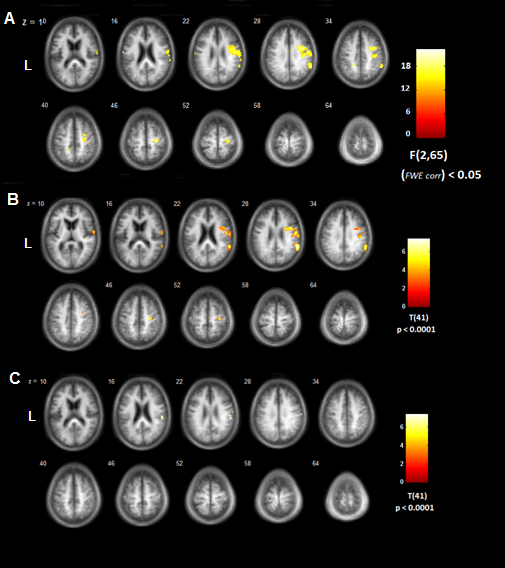
**

Panel A: ANOVA for the complete study population. Panel B: t-test between healthy controls and patients at exam 1. Panel C: t-test between healthy controls and patients at exam 2. Maps are projected onto axial slices of an average anatomical image of the complete cohort (L, left). Coordinates are given in MNI space (mm).

**Table S2. Results of between-group comparison of CBF in healthy controls and stroke patients**

| **Anatomical region** | **Cytoarchitectonic Area (%)*** | **x** | **y** | **z** | **voxels** | **HC-P *F*(*p_FWE_*)°** | **HC-P Month 3 *T*(*p_unc_*)#** | **HC-P Month 9 *T*(*p_unc_*)#** | **rCBF HC** | **rCBF P Month 3** | **rCBF P Month 9** |
| --- | --- | --- | --- | --- | --- | --- | --- | --- | --- | --- | --- |
| *Ipsilesional* |  |  |  |  |  |  |  |  |  |  |  |
| Premotor cortex | Area 6 (60.1) | 38 | -13 | 45 | 918 | 26.05  (< .001) | 5.39  (<.001) | 3.27  (< .01) | 50.4±17.0 | 29.7±22.1 | 31.9±22.2 |
| Parietal operculum | OP1 (11.1) IPC(PFop) (7.1) | 61 | -24 | 18 | 575 | 22.23  (< .01) | 3.49  (<.01) | 3.91  (<.005) | 48.9±16 | 28.8±18.4 | 31.8±17.4 |
| Inferior parietal lobule | IPC (PGa) (71.6) | 56 | -44 | 30 | 409 | 14.21  (< .01) | 5.67  (<.001) | 3.44  (< .01) | 54.8±16.0 | 28.6±18.4 | 31.7±17.4 |
| Post-/Precentral gyrus | Area 4p (40.3) Area 3b (32.0) | 28 | -36 | 61 | 122 | 14.41  (< .01) | 5.2  (<.001) | 2.92  (< .01) | 50.3±18.0 | 21.5±15.5 | 22.0±15.5 |
| *Contralesional* |  |  |  |  |  |  |  |  |  |  |  |
| Inferior Precuneus | n. a. | -15 | -42 | 36 | 136 | 2.02  (< .01) | 1.58  (n.s.) | 3.99  (< .01) | 50.3±20.2 | 41.5±9.5 | 27.6±13.0 |

Abbreviations: CBF, cerebral blood flow, HC, healthy controls; n.a., not assigned. P, patients. FWE-corr and unc, indicate family-wise error corrected or uncorrected p-values. CBF values are in [ml/100g/min].° F-statistic from mixed ANOVA (HC, one measurement; P, two repeated measurements). #T-statistic from post-hoc unpaired t-test (HC versus P at each time point). * Percent of cluster on that area.
