# Supplementary material for: Interhemispheric Cerebral Blood Flow Balance during Recovery of Motor Hand Function after Ischemic Stroke—A Longitudinal MRI Study Using Arterial Spin Labeling Perfusion
Source: PLoS One. 2014 Sep 5;9(9):e106327. doi: 10.1371/journal.pone.0106327 (PMC4156327; doi:10.1371/journal.pone.0106327)
Supplement: Supporting Information S3 — Describes the main findings in interhemispheric balance changes and the detailed LIs according to regions and patient subgroups. (DOCX) [file pone.0106327.s003.docx]

# Supporting information S3

**Global CBF, Signal-to-Noise Ratio, and Reliability**

Mean grey matter CBF values were not significantly different between controls and patients across examinations (mixed-effects ANOVA: F (2, 51) =2.8; p >.05), further indicating that our results could not be explained by a global perfusion reduction in patients. Also, average SNR of CBF maps was not different across measurements (controls: 2.25 ± 1.65, patients' Month 3: 2.15 ± 1.61, and Month 9: 2.14 ± 1.63). Values of global and grey matter CBF were highly correlated across examinations in the longitudinal healthy control group (r=.89 and r = .78, respectively, both p<.01).

**
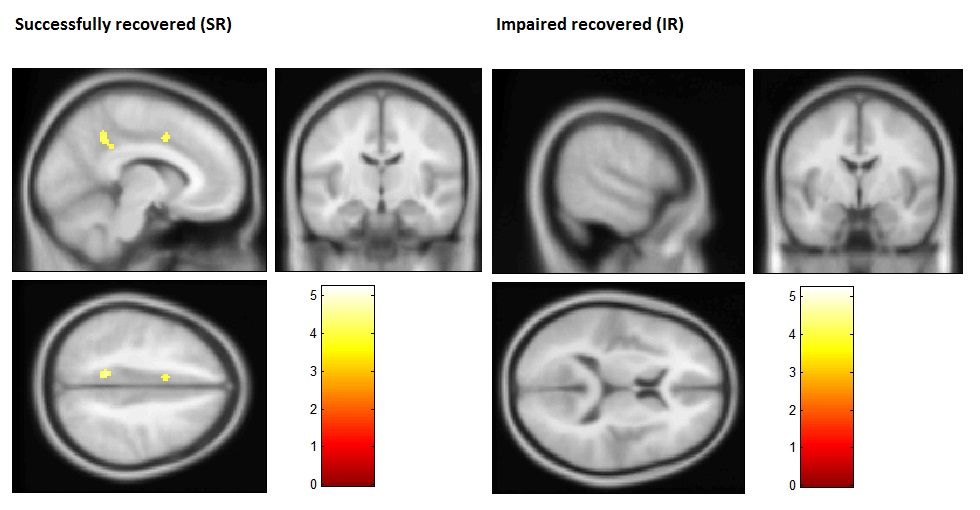
Figure S2 Longitudinal CBF differences between three and nine months for patients with successful and impaired recovery**

This figure shows longitudinal CBF effects after adjustment for total lesion size. Although CBF effects were reduced in both groups, there was a stronger lesion dependence in the IR group. See main text for details.

**Longitudinal changes in laterality indices within the sensorimotor network**

**
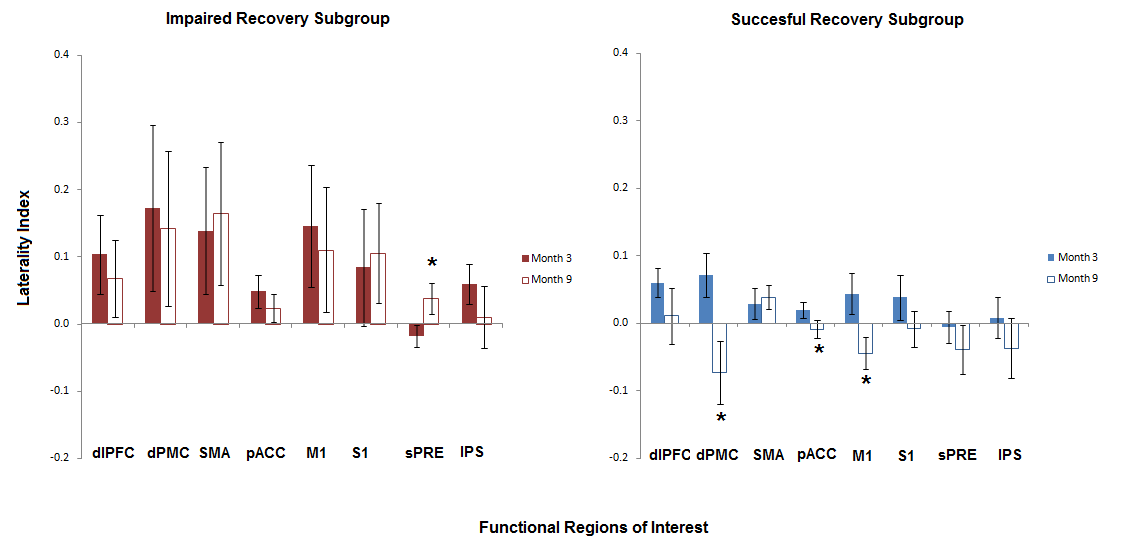
**

Figure S3 summarizes the main findings in interhemispheric balance changes using functional ROI analysis (i.e. nodes of the sensorimotor network, Table S1). Bar plots indicate the mean ± 95% confidence interval for the laterality index in functional nodes (regions-of-interest) of the sensorimotor network. Abbreviations are given in Table S1. Asterisks denote significant change in LI (p<0.05, corrected).
